# A dataset of estimated heterozygous individual and carrier couple frequencies for pan-ancestry carrier screening

**DOI:** 10.1016/j.dib.2025.111835

**Published:** 2025-06-25

**Authors:** Mia J. Gruzin, Matthew Hobbs, Sarah Poll, Swaroop Aradhya, Leslie Burnett

**Affiliations:** a Garvan Institute of Medical Research, Darlinghurst, NSW 2010, Australia; b School of Clinical Medicine, UNSW Medicine and Health, St Vincent’s Clinical Healthcare Campus, Darlinghurst, NSW 2010, Australia; c Labcorp Genetics Inc (formerly Invitae Corporation), San Francisco, CA 94304, USA; d Stanford University School of Medicine, Department of Pathology, Stanford, CA 94304, USA; e Northern Clinical School, Faculty of Medicine and Health, University of Sydney, St Leonards, NSW 2065, Australia; f Virtus Genetics, Virtus Health Specialist Diagnostics, Revesby NSW 2212, Australia

**Keywords:** Reproductive genetics, Carrier screening, Pan-ancestry, Equitable

## Abstract

The data described in this publication supported the development and evaluation of pan-ancestry reproductive carrier screening panels for autosomal recessive (AR) and X-linked (XL) conditions.

Raw data included combined sets of DNA variants in 1,350 AR/XL genes obtained from the ClinVar and gnomAD databases. The dataset enabled calculations of positive yield for individuals and couples across both ancestry-specific and pan-ancestry, optimised "Goldilocks"-ranked gene panels, addressing population-specific variations in the frequencies of heterozygous individuals and carrier couples.

The positive yield analysis offered a performance metric for carrier screening panels, facilitating the modeling of screening performance for panels of varying sizes and composition and providing resources for optimizing panel content to ensure equity across underrepresented genetic ancestries

The dataset can support ongoing research into the equitable application of carrier screening and offers significant reuse potential for refining population genetic screening practices, validating computational models, and developing frameworks to update carrier screening panels in alignment with evolving genomic data, including in underrepresented and minority populations.

Specifications Table

**Table:** 

Subject	Health and Medical Sciences: Clinical Genetics
Specific subject area	Reproductive genetics, bioinformatics, carrier screening, and equitable genomic applications for underrepresented genetic ancestries
Type of data	Table, Processed Data
Data collection	The dataset was generated by integrating ClinVar and gnomAD exome and genome sequencing data tables using bioinformatics pipelines. Genes associated with autosomal recessive (AR) and X-linked (XL) conditions were curated based on clinical significance and population-level allele frequencies. Inclusion criteria focused on genes with established pathogenic variants and relevance to reproductive carrier screening. Data were normalized for population-specific allele frequencies and aggregated to model positive yield for individuals and couples across diverse ancestries.
Data source location	The modeling data presented was collected using Hail Query Python (https://hail.is/docs/0.2/api.html) on Terra on Google Cloud. Joint tables were constructed from two open-source major genetic databases: gnomAD (https://gnomad.broadinstitute.org/), which was used for population data for ten genetic ancestries, and ClinVar (https://www.ncbi.nlm.nih.gov/clinvar/), which was used for human variation data. Data was stored at: Institution: Garvan Institute of Medical Research City: Sydney Country: Australia
Data accessibility	Repository name: Mendeley Data Data identification number: URL: https://doi.org/10.17632/ndhmx93v4v/2 DOI: 10.17632/ndhmx93v4v.2 Direct URL to data: https://data.mendeley.com/datasets/ndhmz93v4v/2
Related research article	M. J. Gruzin, M. Hobbs, R.E. Ellsworth, S. Poll, S. Aguilar, J. Knezovich, N. Faulkner, N. Olsen, S. Aradhya, L. Burnett, Optimizing gene panels for equitable reproductive carrier screening: The Goldilocks approach. Genet. Med. 27 (2025) 101387. https://doi.org/10.1016/j.gim.2025.101387

## 1 Value of the Data

• Comprehensive and current population data for AR/XL Mendelian disorders: This dataset provides population-specific and pan-ancestry gene carrier frequencies for 1,350 genes associated with AR/XL conditions by integrating current ClinVar and gnomAD data. This data supports researchers and laboratories designing carrier screening panels tailored to diverse genetic ancestries.
• Positive yield modeling: Our dataset includes positive yield estimations for heterozygous individuals and carrier couples for carrier screening panels of varying gene compositions in all ten gnomAD PCA-defined ancestry groups, offering researchers a resource to evaluate screening performance and optimize panel content across ancestry groups.
• Equity in genomics: The data addresses disparities in population sample sizes in gnomAD by incorporating data for underrepresented ancestries. We also present data for a simulated population, “ALLGRPMAX”, which represents the maximum gene carrier rate for a gene from any of the ten gnomAD ancestry groups. Ranking genes by their gene carrier rate in ALLGRPMAX promotes genetic equity, enabling the development of equitable screening panels, because each of the high-risk genes from any ancestry group are prioritized in the ranking system. We note we cannot fully account for minority ancestries that are either not present or under-represented in the gnomAD database.
• Reusability in research: Researchers can use the dataset to validate computational models, refine gene panels, and study population-specific genetic carrier rates, fostering advancements in reproductive genetics and bioinformatics.
• Dynamic panel and Guideline updates: The related Research Article and this dataset provide a framework for updating carrier screening panels using evolving genomic data, facilitating continual alignment with and updating of clinical carrier screening guidelines.

## 2 Background

The data presented here is associated with the 2024 article ‘Optimizing Gene Panels for Equitable Reproductive Carrier Screening: the “Goldilocks” Approach’, by Gruzin et al. [1,2].

Reproductive carrier screening is a form of genetic testing offered before or during early pregnancy to identify individuals and/or couples at increased risk of having a child with a severe inherited condition [3]. Reproductive risk arises when both partners in a couple carry pathogenic variants in the same autosomal recessive (AR) gene, or when an XX partner (with a female assigned sex at birth) carries an X-linked (XL) gene. Identifying such couples enables informed reproductive decision-making, including assisted reproductive technologies, prenatal diagnosis, termination or adoption [4].

Historically, carrier screening targeted a small number of conditions in a specific racial, ethnic, or ancestry (REA) group [5]. For example, Tay-Sachs screening traditionally focused on Ashkenazi Jews due to a higher disease incidence in this group compared to the general population [6]. The advent of next-generation sequencing (NGS) technology; however, now allows simultaneous screening of thousands of genes, enabling expanded, pan-ancestry screening approaches [7,8]. This is supported by professional organisations for reducing bias and improving equity in reproductive medicine [[9], [10], [11], [12], [13], [14]].

Despite increased capacity for testing more genes in a carrier screening test, there remains no consensus on which genes should be included. Some suggest using a threshold of the carrier frequency in the population [9,10,15,16], whereas others have suggested prioritising based on other criteria, such as “severity” of the condition and age of onset [[17], [18], [19]]. A 2023 study by Wang *et al.* [20] highlighted wide variability across commercial and clinical panels, with some testing dozens or even thousands of genes.

This dataset underpins our efforts to define effective, equitable gene panels for pan-ancestry carrier screening, using gnomAD v4.1.0 and ClinVar data. Recognizing the historic Eurocentric bias in public genomic datasets [5], we apply an “ALLGRPMAX” strategy (described below) to mitigate underrepresentation of minority ancestry groups and improve equity.

## 3 Data Description

### 3.1 Overview

This article describes the dataset of the linked repository, containing data on the frequency of genetic variants associated with 1,350 AR/XL Mendelian conditions. It includes raw variant-level data and processed gene-level estimates of gene carrier rates (GCRs) for individuals and of gene couples’ carrier rates (GCCR) for couples, stratified by ten ancestry groups. This resource enables researchers to access carrier frequencies for specific genes to predict disease incidence and prevalence within an ancestry group, evaluate and compare candidate gene lists across diverse ancestry groups and develop equitable carrier screening panels (as we have done in [1]).

In our linked study (Gruzin et al*.*, 2025) [1], we used this dataset to develop a series of “optimal” pan-ancestry genetic screening panels that maximize equity and carrier detection while minimizing resources (cost and variant interpretation). We have termed this approach, “Goldilocks”, to reflect the aim of designing carrier tests that are not “too small” (minimizing detection), nor “too big” (requiring additional resources).

### 3.2 Dataset overview

Raw data files include variant allele frequencies (AF) for ClinVar-classified Pathogenic/Likely Pathogenic (“reportable”) variants in 1,350 genes. Variant AFs were extracted from different gnomAD versions (gnomAD v2.1.2, v3.1.1., v4.1.0) using exome and genome sequencing data. Frequencies were collected for the entire gnomAD dataset ("ALL") and across ten ancestry groups defined by Principal Component Analysis: African/African American (AFR), Amish (AMI), Admixed American (AMR), Ashkenazi Jewish (ASJ), East Asian (EAS), Finnish (FIN), Middle Eastern (MID), non-Finnish European (NFE), South Asian (SAS), and Remaining Individuals (RMN). Variant lists were downloaded from ClinVar, and filtered to include only previously reported reportable variants. Variants with conflicting interpretations of pathogenicity, variants of uncertain significance and variants classified as Benign/Likely Benign were excluded. The gene list (n=1,350) was constructed by combining the gene list used by the American College of Medical Genetics and Genomics in 2021 [9,10,15] with expert-curated gene lists by Mackenzie’s Mission [17].

Processed data applies a binomial-like modeling to estimate individual carrier rates (GCR) and couples’ carrier rates (GCCR) for each gene. Carrier couples were defined as two individuals from the same genetic ancestry group. This was calculated separately for each ancestry group using principles based on the Hardy-Weinberg Equilibrium and can be used to model population-level cumulative carrier risk for individuals and couples.

### 3.3 File formatting and organization

An overview of the dataset structure, organized by file name, is provided in Table 1. Files follow a standardized naming convention to distinguish between **raw data (labelled with the prefix 1n)** and **processed data (labelled with the prefix 2n)**, where “n” is an alphabetical suffix in the range [a..l].

**Table 1:** Overview of dataset in the linked repository. Files labelled with a prefix of 1n (where “n” is an alphabetical suffix) are raw combined gnomAD and ClinVar data for 1,350 genes. The modeling files are labelled with a prefix 2n (where “n” is an alphabetical suffix), and have been filtered by ClinVar clinical review status and contain gene carrier rates for individuals (GCR) and couples (GCCR) and positive yield data for ancestry-specific and pan-ancestry carrier screening gene lists. Joint data refers to both combined exome and genome data.

File name	gnomAD version	gnomAD dataset	Dataset sample size	Number of genes
1a GL_Terra_output_gnomADv2_exomes.tsv	V2.1.2	Exomes	125,748	1,261
1b GL_Terra_output_gnomADv2_genomes.tsv	V2.1.2	Genomes	15,708	1,081
1c GL_Terra_output_gnomADv3_genomes.tsv	V3.1.1	Genomes	76,156	1,244
1d GL_Terra_output_gnomADv4_joint.tsv	V4.1.0	Joint	807,162	1,330
2a GL_ALLGRPMAX_gnomADv2_exomes_NO_STAR.tsv	V2.1.2	Exomes	125,748	1,261
2b GL_ALLGRPMAX_gnomADv2_exomes_ONE_STAR.tsv	V2.1.2	Exomes	125,748	1,237
2c GL_ALLGRPMAX_gnomADv2_exomes_TWO_STAR.tsv	V2.1.2	Exomes	125,748	1,066
2d GL_ALLGRPMAX_gnomADv2_genomes_NO_STAR.tsv	V2.1.2	Genomes	15,708	1,081
2e GL_ALLGRPMAX_gnomADv2_genomes_ONE_STAR.tsv	V2.1.2	Genomes	15,708	1,054
2f GL_ALLGRPMAX_gnomADv2_genomes_TWO_STAR.tsv	V2.1.2	Genomes	15,708	855
2g GL_ALLGRPMAX_gnomADv3_genomes_NO_STAR.tsv	V3.1.1	Genomes	76,156	1,244
2h GL_ALLGRPMAX_gnomADv3_genomes_ONE_STAR.tsv	V3.1.1	Genomes	76,156	1,212
2i GL_ALLGRPMAX_gnomADv3_genomes_TWO_STAR.tsv	V3.1.1	Genomes	76,156	1,305
2j GL_ALLGRPMAX_gnomADv4_joint_NO_STAR.tsv	V4.1.0	Joint	807,162	1,261
2k GL_ALLGRPMAX_gnomADv4_joint_ONE_STAR.tsv	V4.1.0	Joint	807,162	1,310
2l GL_ALLGRPMAX_gnomADv4_joint_TWO_STAR.tsv	V4.1.0	Joint	807,162	1,147

Files labeled **1n** are in TSV format and contain raw variant allele frequency data for reportable variants. These files were generated from gnomAD and ClinVar data using Hail Query in Python. The header fields include variant identification (including “locus,” “alleles,” “clinvar_id,” and “geneinfo”); variant classification data (clinical significance in ClinVar [“clnsig”], “variant_type,” and “molecular_consequence”) and frequency data (allele number [“an”], allele count [“ac”], allele frequency [“af”], and homozygote count [“hom_count”] for XX, XY, and combined XX/XY samples in ALL and in each ancestry group). Additionally, frequency data for “grpmax” (previously known as “popmax”) is included, which records the an/ac/af/hom_count values for the non-bottlenecked ancestry group with the highest allele frequency, excluding ASJ, FIN, and RMN groups.

Files labelled **2n** are Excel files containing multiple sheets of processed modeling data that evaluate positive yield across ancestry-specific and pan-ancestry populations. These files enable comparisons of GCR and GCCR for individuals and couples, respectively, and cumulative yields of increasingly sized gene panels. The ranking of genes in each sheet follows the descending order of their GCCR, facilitating the prioritization of genetic screening panels by highlighting genes with the highest risk to the couple. The first sheet in each file, labeled “Summary,” presents graphs and tables that illustrate the positive yield of a pan-ancestry ("allgrpmax") panel applied to each of the ten gnomAD ancestry groups. The term "allgrpmax" follows the same logic as "grpmax" but does not exclude bottlenecked groups, considering the highest allele frequency in any of the gnomAD groups.

Subsequent sheets are labeled “ALLGRPMAX,” “ALL,” and the ten ancestry groups. The “ALLGRPMAX” sheet contains the gene list and associated positive yield for an increasingly sized allgrpmax panel, ranked in descending GCCR order within allgrpmax. The other sheets display the positive yield for both an allgrpmax panel and an ancestry-specific panel applied to the specified ancestry group, enabling direct comparisons between targeted ancestry-specific and pan-ancestry gene panels. Apart from the “Summary” and “ALLGRPMAX” sheets, the header fields in these files include the gene name, individual and couple carrier frequency (cf, ccf), non-carrier frequency (non_cf, non_ccf), cumulative non-carrier frequency (cum_non_cf, cum_non_ccf), cumulative carrier frequency (cum_cf, cum_ccf), and positive yields for individuals and couples (positive_yield_ind, positive_yield_couple). The gene order is specified as either allgrpmax order (also referred to as Goldilocks order) or sheet order. If not explicitly stated as sheet order, the header fields are applied in allgrpmax order by default.

## 4 Experimental Design, Materials and Methods

This study used de-identified, aggregated data from publicly available genomic repositories, including gnomAD and ClinVar.

**Fig. 1:**
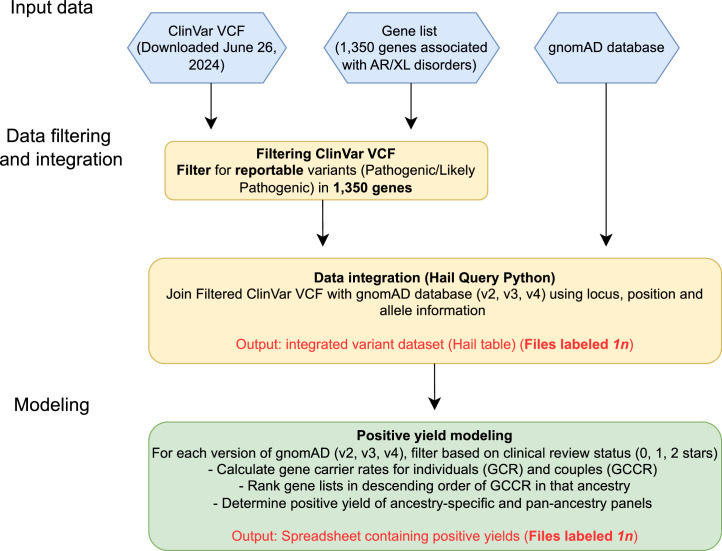
Overview of Methods Workflow. Input files are indicated in blue hexagons. Processing steps are indicated in yellow and green boxes. Output files are shown as red text. ClinVar variant call format (VCF) files annotated both to GRCh37 and GRCh38 were downloaded on June 26, 2024, and filtered for reportable variants in specific curated gene lists. ClinVar and gnomAD data was combined and downloaded using Hail Query Python. Resultant output files were filtered for ClinVar clinical review status (CLNREVSTAT), and modeling was performed to determine the probability of being a heterozygous individual (gene carrier rate [GCR]) and carrier couple (gene couples’ carrier rate [GCCR]) for a given gene in each ancestry group. Cumulative positive yield of being a heterozygous individual or carrier couple across multiple genes was then calculated for ancestry-ranked and pan-ancestry gene lists.

An overview of the experimental design is summarized in Fig. 1. This describes the workflow, starting with raw ClinVar and gnomAD datasets, and ending with processed modeling data used to estimate positive yield of testing. Positive yields are based on GCR and GCCR, which represent the probability of being a heterozygous individual or a carrier couple in each gene, respectively.

### 4.1 Preparation of datasets

ClinVar variant call format (VCF) files separately annotated to both GRCh37 and GRCh38 were extracted on June 26, 2024. The gnomAD datasets extracted included the exome and genome sequencing data in version v2.1.1 (“v2”), genome data in v3.1.2 (“v3”), and joint exome and genome data in v4.1.0 (“v4”).

Data parsing and bioinformatic analyses began with filtering the ClinVar VCF files for reportable variants (clinical significance was “Pathogenic” and/or “Likely Pathogenic”, without conflicting interpretations of pathogenicity) in specific curated gene lists. We first considered 1,350 genes derived from multiple sources, including the 415 genes analyzed by Guo and Gregg [3,4], the ACMG Tier 3 list [5], and the Mackenzie’s Mission v2.1 list [6]. This comprehensive gene list formed the basis for subsequent analyses for the “Goldilocks” approach described in [1] to determining the optimal number of genes to include for equitable carrier screening.

The Hail Query Python API on the Terra platform was employed to combine ClinVar data with allele frequency data from gnomAD. Ancestry groups within gnomAD were defined based on Principal Component Analysis (PCA)-derived classifications and included ten distinct groups: African/African American (AFR), Amish (AMI), Admixed American (AMR), Ashkenazi Jewish (ASJ), East Asian (EAS), Finnish (FIN), Middle Eastern (MID), non-Finnish European (NFE), South Asian (SAS), and Remaining Individuals (RMN). When referring to the entirety of gnomAD’s aggregated samples, the term “ALL” was used.

For certain conditions, where the mechanism of disease was not accurately captured in our gnomAD/ClinVar analysis, including Fragile X Syndrome and Spinal Muscular Atrophy, allele frequency data from the literature (Invitae Corporation, San Francisco, CA) were used in place of gnomAD estimates [7].

To construct the modeling datasets, variants were further filtered based on ClinVar’s clinical review status (“CLNREVSTAT”), where: 0-star CLNREVSTAT indicated the variant was submitted without assertion criteria or evidence, 1-star indicated the variant was submitted by only one laboratory and 2-star indicated the variant had multiple submitters, without conflicts. Variants were excluded if they had an allele frequency greater than 0.05 in any ancestry group.

### 4.2 Modeling gene carrier rates

We first calculated the probability of having at least one pathogenic germline variant (PGV) (P_i_) in each modeling group (gnomAD ALL and each constituent ancestry group):

$$P_{i}=1-\prod_{j=1}^{n}\left(1-PGV_{j}\right)$$

Where *n* was the number of pathogenic variants in the gene and *PGV* was the variant allele frequency.

We then modeled the probability of being a heterozygous individual (gene carrier rate [GCR]) for AR and XL genes, based on Hardy-Weinberg equilibrium:

$$GCR_{AR_{i}}=2*p*q=2*\left(1-q\right)*q$$

$$GCR_{XL_{i}}=p*q=\left(1-q\right)*q$$

Where *GCR* was calculated for gene “*i*", *p* (wild-type gene allele frequency) was calculated as the complement of *q (*disease-causing gene allele frequency, Pi), *GCR_ARi_* represents any tested individual and *GCR_XLi_* represents an XX individual, given that hemizygous males would presumably be affected and seek diagnostic testing, not carrier screening. For XL conditions, *q* was extracted from the XX-samples only (excluding XY samples).

We then estimated the probability of being a carrier couple (gene couples’ carrier rate [GCCR]). When we refer to a carrier couple, we use it to mean a couple where both reproductive partners are heterozygous for a pathogenic variant in the same AR gene, or if the XX partner is heterozygous for a pathogenic variant in an XL gene. Couples were defined as a reproductive union between an XX and an XY individual from the same ancestry group. For AR conditions, both individuals were required to be heterozygous for a reportable variant in the same gene, whereas for XL conditions, only the XX individual was required to be heterozygous:

$$GCCR_{AR_{i}}=GCR_{AR_{i}}*GCR_{AR_{i}}=4*{\left(1-q\right)}^{2}*q^{2}$$

$$GCCR_{XL_{i}}=GCR_{XL_{i}}*p={\left(1-q\right)}^{2}*q$$

### 4.3 Evaluating Goldilocks points

Using the comprehensive list of 1,350 genes, positive yields for carrier screening panels were then calculated for varying panel sizes, with thresholds established at 90%, 95%, 99%, and 99.7% yield levels. Gene panels that achieved these positive yield thresholds across all ancestry groups, denoted as N_90_, N_95_, N_99_, and N_99.7_, were defined as the “Goldilocks” panels. These gene lists are provided in Gruzin et al. [1].

## Limitations

There are several limitations to our study. Our model is based on Hardy-Weinberg equilibrium, assuming large population size, random mating, and the absence of new variants. It does not account for asymmetric inheritance patterns. Some genes in our model, such as *DHODH, ANKS6, F8, TNNT1, TMCO1, C6* and *C8B*, are often absent from current commercial carrier screening panels due to limitations in standard short-read next-generation sequencing [8]. Our analysis is restricted to ClinVar variants present in gnomAD, which currently primarily includes SNVs and small indels. We have assumed all modeled ClinVar variants have been correctly classified, and that the testing laboratory is capable of detecting all variants. Additionally, gnomAD v4 PCA is limited to ten genetic ancestry groups, despite more diverse and complex ancestries in the human population. These databases also have a bias, disproportionately representing European genomic data, which our ALLGRPMAX approach corrects, but cannot fully compensate if the relevant minority ancestry group is absent from the database [5]. Furthermore, our model assumes couples consist of two individuals within the same specific ancestry group, while this constraint does not exist in the real world. Finally, the datasets we used did not provide an individual-level identifier variable due to privacy reasons, therefore the empirical cumulative GCR was unavailable.

## Ethics Statement

The authors have read and follow the ethical requirements for publication in Data in Brief and confirming that the current work does not involve human subjects, animal experiments, or any data collected from social media platforms.

## Credit Author Statement

Conceptualization: L.B.; Data Curation and Formal Analysis: M.J.G; Investigation, Methodology and Software: M.J.G., M.H., L.B.,; Project Administration and Supervision: Sw.A., L.B.; Visualization: M.G.; Writing- original draft: M.J.G, L.B.; Writing- review and editing: M.J.G, S.P., Sw.A., L.B.

## Data Availability

Mendeley DataA dataset of estimated heterozygote and carrier couple frequencies for pan-ancestry carrier screening. (Original data).
